# Screening for loneliness in representative population samples: Validation of a single-item measure

**DOI:** 10.1371/journal.pone.0279701

**Published:** 2023-03-16

**Authors:** Anna Celine Reinwarth, Mareike Ernst, Lina Krakau, Elmar Brähler, Manfred E. Beutel

**Affiliations:** 1 Department of Psychosomatic Medicine and Psychotherapy, University Medical Center, Johannes Gutenberg-University Mainz, Mainz, Germany; 2 Department of Psychiatry and Psychotherapy, Medical Faculty, University of Leipzig, Leipzig, Germany; Kyung Hee University School of Medicine, REPUBLIC OF KOREA

## Abstract

**Background:**

Loneliness is a highly relevant public mental health issue. This work presents the validation of a single-item measure of loneliness and its subjective experience: “I am frequently alone/have few contacts”. It can be used in large-scale population surveys where an economical assessment is of key importance.

**Methods:**

Data was drawn from two representative German population surveys conducted in early and late 2020 (combined *N* = 4,984; 52.9% women; age: *M* = 48.39 years (*SD* = 17.88)). We determined the prevalence of loneliness in men and women across different age groups. In order to test concurrent validity, bivariate correlation analyses and Chi-square tests were performed. Convergent and discriminant validity were tested by investigating intercorrelations of the single-item measure of loneliness with another loneliness measure, other mental health outcomes, and associations with sociodemographic characteristics.

**Results:**

Based on the single-item measure, 23.4% of participants reported some degree of loneliness, 3.4% among them severe loneliness. Comparisons with the LS-S showed similar prevalence rates of loneliness. A moderately positive relationship between the two loneliness measures was found by bivariate correlation analysis (*ρ* = .57, *p* < .001), but results indicated only weak convergent validity. Construct validity was supported by associations with depressive symptoms, anxiety symptoms, satisfaction with life, household size, and partnership.

**Conclusions:**

Loneliness is frequently reported in the general population. The single-item measure of loneliness is suitable as a brief screening measure in population-based assessments.

## Introduction

In recent years, loneliness has been acknowledged as a consequential public health issue and has increasingly come to the fore of research and policy considerations due to its widespread negative implications for mental and physical health outcomes [1, 2]. Several studies indicate that loneliness is more prevalent among certain vulnerable groups of the population, for instance, young people or the oldest old [2–4]. Most recently, loneliness has also been discussed as a major psychosocial consequence of the COVID-19 pandemic [5, 6] as ongoing restrictions of social contacts promote social isolation [7], which in turn is a strong risk factor for loneliness [8].

Loneliness has been defined as the emotional response to a perceived discrepancy between the desired quantity and quality of social life with one’s actual social relationships [9, 10]. Feeling lonely is a subjective, distressing experience [11], which matters, especially for mental health. Many international research efforts are undertaken to deepen our understanding of the underlying societal and personal risk constellations, with the aim to develop prevention and intervention programs [12, 13].

Recent findings indicate that about 10–15% of adults often feel lonely [9, 14]. In the context of the COVID-19 pandemic, continuous loneliness values as well as prevalence rates have further increased around the world, as shown by a recent systematic review with meta-analysis summarizing the most recent international research [5]. Large population-based studies indicated an increase in loneliness in the German population in the context of the COVID- 19 pandemic to as high as 32% [15, 16].

Several sociodemographic characteristics such as age, sex, partnership, household size and income; subjective and functional health, and cognitive skills have been considered as risk or protective factors, respectively, on an individual level [17, 18]. Especially young and old adults were likely to suffer from higher levels of loneliness [4, 14, 19], with rising numbers seen especially in emerging adulthood [3, 15, 16]. Findings regarding sex differences were inconsistent: Some studies found higher degrees of loneliness among women compared to men across age groups [9, 14–16, 20, 21], while others reported the opposite [22, 23], or did not observe sex differences in mean levels of loneliness [24].

As Nicolaisen and Thorsen [22] pointed out, different prevalence rates may be the result of the heterogeneity of loneliness measures, i.e. more direct and indirect measures. The direct way of measuring loneliness is through e.g., single items that ask explicitly whether a person has felt lonely, including the specific word “lonely” or “loneliness”. The use of multiple-item scales represents an indirect way of capturing loneliness. The UCLA Three-item loneliness scale [25] and the De Jong Gierveld Loneliness Scale [26] are two of the most widely used indirect measures of loneliness. Both consist of multiple indirect questions related to the subjective evaluations of one´s social relationships and they do not include the word “lonely” or “loneliness”. A comparison of the prevalence rates of loneliness as indicated by direct and indirect measures showed higher levels of loneliness in women when using a more direct measure, while loneliness was more prevalent among men when using an indirect measure [22].

The construct of loneliness lends itself to the assessment via a single-item measure as it is unambiguous and narrow in scope [27, 28]. Those which were validated showed good psychometric properties in recent research [29]. However, not all single-item measures of loneliness used in surveys have previously been validated. The widespread use of untested and unstandardized instruments (e.g., adapted from questionnaires used to assess different constructs such as depression) hampers the quality and meaningful integration of international, original research [5].

In order to provide a single-item measure of loneliness as a suitable and economic alternative to previous loneliness scales, especially for large-scale surveys, the purpose of the present study was to validate a single-item measure screening for loneliness that could be used in large-scale research projects. This measure was previously created for use in the German population-representative Gutenberg Health Study (GHS) [14]. Participants were asked to both report their level of agreement with the statement “I am frequently alone/have few contacts” as well as how much they suffered (in the case of agreement). Thus, as the statement directly addresses the extent of contact “alone, few contacts” and the subjective experience of loneliness, the single-item measure constitutes a more direct measurement of loneliness. It has previously been associated with relevant health variables such as depression and anxiety symptoms and health behavior [14, 30, 31]. Also, it has statistically predicted suicidal ideation and anxiety symptoms [20] as well as depression symptoms over time [32, 33]. Yet, a thorough validation of the single-item measure of loneliness which includes the investigation of associations with established instruments such as the German version of the UCLA Three-item loneliness scale [LS-S; 34] has been lacking.

Specifically, we aimed to:

1. determine the degree of loneliness in men and women across different age groups in a combined sample of two representative surveys in early 2020 (survey A) and late 2020/early 2021 (survey B) based on the single-item measure of loneliness
2. assess convergent validity by comparing the overall prevalence of loneliness based on the single-item measure of loneliness and the LS-S (survey A)
3. investigate the construct validity of the single-item measure of loneliness by examining associations with other mental health outcomes (depression symptoms, anxiety symptoms, satisfaction with life), and previously established sociodemographic risk factors (household size, partnership) (survey A).

## Methods and materials

### Survey procedure

For this study, we used data from two nationwide, representative (with respect to age, sex, and level of education) German population surveys using the same methodology. They were carried out as face-to-face household surveys by trained interviewers of the independent market research institute USUMA. Survey A was conducted between April and June 2020, and survey B between December 2020 and March 2021. Participating households were selected using a random route procedure and Kish selection [35]. The inclusion criteria for participating individuals were an age of ≥ 14 years in survey A and of ≥ 16 in survey B. To match the two surveys’ age range, *n* = 33 participants < 16 years in survey A were excluded from the analyses. Eligibility criteria were an adequate understanding of the German language and provision of verbal informed consent (after being informed about the study procedures, data collection, and anonymization of personal data) which was recognized by the interviewers. The institutional ethics review board of the University Leipzig accepted the study contents and procedures, including the consent procedure (numbers: 002/20-ek (Survey A); 043/20-ek (Survey B)). Moreover, the studies adhered to the guidelines of the ICC/ESOMAR International Code of Marketing and Social Research Practice in addition to ICH-GCP-guidelines.

### Participants

We combined data from surveys A and B, resulting in a total sample of *N* = 4,984 with 47.1% men and 52.9% women with a mean age of 48.39 years (*SD* = 17.88). A small number of participants who did not identify as either male or female was excluded from the analyses (survey A: *n* = 1; survey B: *n* = 4). Sample characteristics of the total sample are depicted in Table 1.

**Table 1:** Sample characteristics of the overall population (N _total_ = 4,984).

Sociodemographic information	*N*	%
Age (*M*, *SD*)	48.4 (17.9)	
Age group		
*16–24 years*	572	11.5
*25–34 years*	766	15.4
*35–44 years*	777	15.6
*45–54 years*	884	17.7
*55–64 years*	935	18.8
*65–74 years*	675	13.5
*≥ 75 years*	375	7.5
Education		
*High school degree*	1,312	26.5
*No high school degree*	3,646	73.5
Employment		
*Employment*	2,883	58.3
*Civil service*, *maternity leave*, *homemaker*	165	3.3
*Unemployed/ short-time work*	298	6.0
*Retired*	1,229	24.9
*In training*	366	7.4
Equivalent income in euros (*M*, *SD*)	2,017	1,011.6
**Living situation**	*N*	%
Marital status		
*Married/ living together*	2,055	41.4
*Married/ not living together*	134	2.7
*Unmarried*	1,713	34.5
*Divorced*	666	13.4
*Widowed*	392	7.9
Partnership		
*Yes*	2,905	59.8
*No*	1,950	40.2
Number of persons in household (*M*, *SD*)	2.2 (1.1)	

*Note*. *M* = Mean; *SD* = Standard deviation.

### Questionnaires

Loneliness was rated on the single-item measure of loneliness: “I am frequently alone/have few contacts” as 0 = “no, does not apply”, 1 = “yes, it applies, but I do not suffer from it”, 2 = “yes, it applies, and I suffer slightly”, 3 = “yes, it applies, and I suffer moderately”, 4 = “yes, it applies, and I suffer strongly” in both surveys. In order to summarize participants’ responses, loneliness was recoded by combining 0 and 1 to indicate “no loneliness or distress”, 2 = “slight loneliness”, 3 = “moderate loneliness”, and 4 = “severe loneliness” in line with a previously established procedure [14]. We also created a binary variable on this basis, combining responses 0 and 1 to indicate “no loneliness”, and 2–4 to indicate “loneliness”.

In survey A, the German Version of the UCLA Three-item loneliness scale, the loneliness scale-SOEP [LS-S] [34] was included as an additional, previously established measure of loneliness. It assesses basic aspects of the subjective experience of loneliness, characterized by the absence of companionship, rejection by peer groups, and feelings of social isolation. Participants indicate on a five-point Likert scale how often such a feeling occurred: 0 = “never”, 1 = “rarely”, 2 = “sometimes”, 3 = “often”, 4 = “very often”. Answers are summarized to a sum score which ranges from 0 to 12, with higher scores indicating higher levels of loneliness [34]. The LS-S showed satisfactory internal consistency [α = .85] and has previously shown measurement invariance with respect to age and sex in a representative German population sample [21]. Besides the sum score, we also created a binary variable. In agreement with the procedures of earlier studies [e.g., 36], we used scores in the top quintile to define loneliness, i.e., this binary loneliness variable indicates a sum score ≥ 5.

The 2-item Patient Health Questionnaire [PHQ-2] measuring core depressive symptoms of depressed mood and anhedonia and the 2-item Generalized Anxiety Disorder Screener [GAD-2] measuring anxiety symptoms [nervousness and worrying] [37] were assessed in surveys A and B. These two measures start with the question: “Over the last two weeks, how often have you been bothered by the following problems?”. The response options ranged from 0 = “not at all” to 3 = “nearly every day”, added to sum scores ranging from 0 to 6. Sum scores of ≥3 for PHQ-2 and GAD-2 were suggested as cut-off points between healthy participants and probable cases of depression or anxiety [38–40]. PHQ-2 and GAD-2 showed acceptable internal consistencies (α = .75, and α = .82) in a large general population sample [37].

Satisfaction with life was measured in survey A using the Questionnaire on Life Satisfaction (Fragebogen zur Lebenszufriedenheit FLZ) [41]. The FLZ measures life satisfaction in eight different domains: Friends and acquaintances, hobbies and leisure, health, financial security, job, housing, family life and children, partnership and sexuality. Participants rate how satisfied they are regarding each domain on a five-point Likert scale: 1 = “not at all satisfied” to 5 = “extremely/very satisfied”. For the single items of the mental health measures that were included in the survey see S1 File.

Sociodemographic characteristics included sex as male, female and diverse, age in years, marital status categorized as married and living together, married and not living together, unmarried, divorced, and widowed, partnership [yes/no], household size in number of persons, education [high school degree/no high school degree], employment categorized as employment, civilian service/ maternity leave or homemaker, unemployed/ short-time work, retired, and in training, and household income in categories (13 categories overall, starting with <500€, 500–649€, 650–749€, 750–750€ and up to >5,000€) in surveys A and B, the mean of each interval was used for further calculation. According to the OECD, we calculated equivalized income as household income/√(people in household) [42].

### Data analysis

Descriptive statistics were computed for each survey and the combined sample. In order to determine the degree of loneliness in 2020, we analyzed the prevalence of loneliness in men and women across different age groups using the combined sample (*N* = 4,984). Due to the absence of the LS-S in survey B, we used only the sample of survey A (*N* = 2,469) to assess concurrent validity in a third step. Bivariate correlation analysis [43] and chi-square tests [44] were used for comparing both measures of loneliness. Additionally, to test convergent and discriminant validity, intercorrelations of the single-item measure of loneliness with PHQ-2, GAD-2, FLZ, and associations with sociodemographic risk factors were investigated in the same sample as used to determine concurrent validity (*N* = 2,469).

All sample characteristics are reported as numbers and percentages or means and standard deviations. All reported *p*-values are based on two-tailed tests. Due to non-normal distribution of the present data, intercorrelations were analysed as Spearman correlations, and associations between two nominal variables were investigated using Chi-square tests. For all data analyses, the software R version 4.1.1 (with the packages haven, psych, and car) was used.

## Results

### Prevalence of loneliness based on the single-item measure

Using the overall sample of 4,984 individuals, we determined the degree of loneliness in the general population in 2020 and early 2021. The majority reported no degree of loneliness or no distress, feeling lonely according to the single-item measure of loneliness (76.5%), while 11.9% reported slight, 8.1% moderate, and 3.4% severe levels of loneliness. The highest prevalence of severe loneliness was found in the age group of 25 to 34 years (4.2%), followed by the age group older than 74 years (4.0%). Severe loneliness was more frequent in women than in men (4.3% vs. 2.5%). Women in the age group older than 74 reported the highest rates of severe loneliness, while men reported the highest rates in the age group of 45 to 54 years.

**Fig 1:**
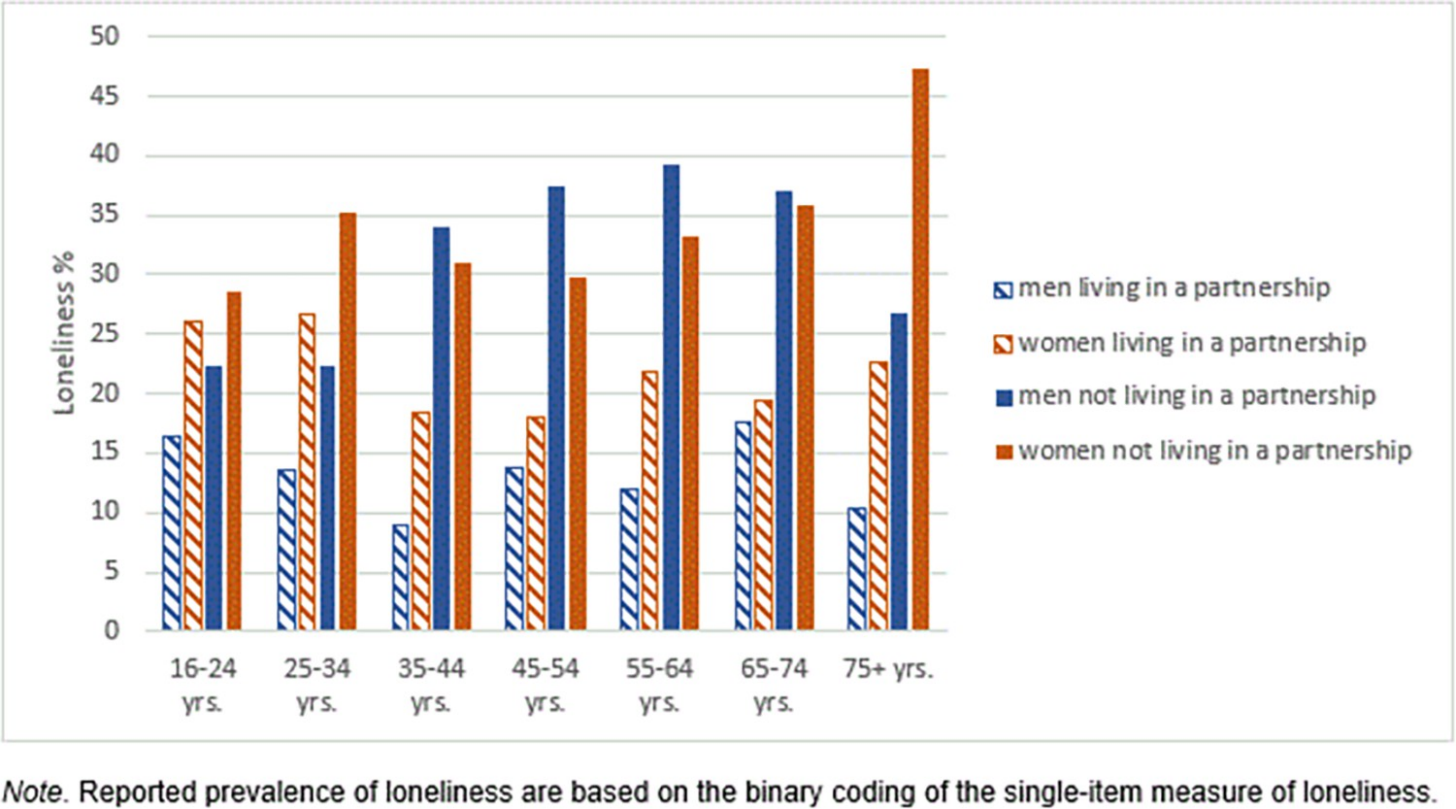
Loneliness across age groups based on a single-item screening measure, stratified by sex and living in a partnership. Reported prevalence rates of loneliness based on the binary coding of the single-item measure of loneliness.

Fig 1 depicts the prevalence of slightly to severely lonely men and women across age groups stratified by partnership. Overall, loneliness was considerably lower among those living in a partnership (17.2% vs. 32.7%); 13% of men and 21.2% of women living in a partnership compared to 30% of men and 34.3% of women not living in a partnership reported some degrees of loneliness. Among women not living in a partnership, loneliness was highly prevalent in the oldest age group [47.4%], while men not living in a partnership reported the highest rates of loneliness in the age group of 55 to 64 years (39.2).

Proportions of lonely individuals, based on the binary coding of the single-item measure of loneliness in the overall sample are presented stratified by sex and age in Table 2.

**Table 2:** Proportions of lonely individuals in the combined survey samples (assessed using the single-item measure), stratified by sex and age group.

**Total** (*N* = 4,956)							
Age group	16–24	25–34	35–44	45–54	55–64	65–74	75+
	(*n* = 570)	(*n* = 763)	(*n* = 772)	(*n* = 878)	(*n* = 929)	(*n* = 672)	(*n* = 372)
	*n* (%)	*n* (%)	*n* (%)	*n* (%)	*n* (%)	*n* (%)	*n* (%)
Loneliness	142 (24.9)	176 (23.1)	151 (19.6)	187 (21.3)	222 (23.9)	173 (25.7)	112 (30.1)
**Men** (*N* = 2,332)							
Age group	16–24	25–34	35–44	45–54	55–64	65–74	75+
	(*n* = 264)	(*n* = 387)	(*n* = 356)	(*n* = 396)	(*n* = 456)	(*n* = 330)	(*n* = 143)
	*n* (%)	*n* (%)	*n* (%)	*n* (%)	*n* (%)	*n* (%)	*n* (%)
Loneliness	55 (20.8)	66 (17.1)	58 (16.3)	82 (20.7)	97 (21.3)	78 (23.6)	22 (15.4)
**Women** (*N* = 2,624)							
Age group	16–24	25–34	35–44	45–54	55–64	65–74	75+
	(*n* = 306)	(*n* = 376)	(*n* = 416)	(*n* = 482)	(*n* = 473)	(*n* = 342)	(*n* = 229)
	*n* (%)	*n* (%)	*n* (%)	*n* (%)	*n* (%)	*n* (%)	*n* (%)
Loneliness	87 (28.4)	110 (29.3)	93 (22.4)	105 (21.8)	125 (26.4)	95 (27.8)	90 (39.3)

*Note*. Reported proportions of lonely individuals based on the binary coding of the single-item measure of loneliness.

### Convergent validity

We used survey A to determine the convergent validity of the single-item measure of loneliness. Comparisons of overall prevalence rates of loneliness calculated based on the single-item measure of loneliness and the items of the LS-S and total scale score showed great similarities: The majority of the participants felt no degrees of loneliness on the single-item measure of loneliness (76.5%), likewise on the LS-S (74.9%). The mean score of loneliness on the LS-S was 3.06 (*SD* = 2.53). On the single-item measure of loneliness, 23.5% of participants reported loneliness, while 25.1% of participants indicated loneliness on the LS-S. For more detailed information about proportions on the single-item measure of loneliness and the three items of the LS-S, see Table 3. Results of bivariate correlation analysis indicated a moderately positive relationship between the single-item measure of loneliness and LS-S score (*ρ* = .57, *p* < .001) and varied across the scale’s three items (absence of companionship: *ρ* = .55, *p* < .010; rejection by peer group: *ρ* = .40, *p* < .010; feelings of social isolation: *ρ* = .45, *p* < .010).

**Table 3:** Comparison of the two loneliness measures included in the study: Single-item measure of loneliness and LS-S.

Single-item measure of loneliness	No loneliness, *n* (%)	Slight loneliness, *n* (%)	Moderate loneliness, *n* (%)	Severe loneliness, *n* (%)	
*Frequently alone/ Few contacts*	1,888 (77.0)	278 (11.3)	200 (8.2)	87 (3.5)	
LS-S	Never, *n* (%)	Rarely, *n* (%)	Sometimes, *n* (%)	Often, *n* (%)	Very often, *n* (%)
*Absence of companion-ship*	524 (21.3)	945 (38.4)	765 (31.1)	172 (7.0)	56 (2.3)
*Rejection by peer group*	848 (34.7)	904 (37.0)	491 (20.1)	159 (6.5)	44 (1.8)
*Feelings of social isolation*	1,399 (57.2)	565 (23.1)	300 (12.3)	130 (5.3)	50 (2.0)

*Note*. LS-S = Loneliness Scale-SOEP.

Using the respective binary coding of the single-item measure of loneliness and the LS-S, we further determined the association of both measures. Chi-square test results indicated dependency (χ^2^(1) = 775.26, *p* < .001, *φ* = .57): Of the 561 participants classified as lonely based on the single-item measure of loneliness, 390 individuals were also identified as lonely based on the LS-S. Of the 1,866 individuals who were not lonely according to the single-item measure of loneliness, 1,651 were also classified as not lonely based on the LS-S’s cut-off, yielding comparable proportions.

#### Construct validity

In order to determine the construct validity of the single-item measure of loneliness, we investigated its intercorrelations with depression symptoms, anxiety symptoms, satisfaction with life, and its associations with previously established demographic risk factors for loneliness (household size, partnership), using survey A (*N* = 2,469). Between the single-item measure of loneliness and PHQ-2 scores, and GAD-2 scores, we observed moderately positive Spearman correlations of *ρ* = .41, and *ρ* = .34. Moderately negative Spearman correlations were found for each domain-specific FLZ satisfaction scores: *ρ* = -.21, friends and acquaintances; *ρ* = -.20, hobbies and leisure; *ρ* = -.22, health; *ρ* = -.24, financial security; *ρ* = -.24, job; *ρ* = -.18, housing; *ρ* = -.23, family life and children; *ρ* = -.28, partnership and sexuality. All of these correlations were highly significant (*p* < .001). Household size and partnership as sociodemographic risk factors for loneliness showed a small correlation of *ρ* = -.13, *p* < .001; χ^2^(3) = 173.29, *p* < .001, *V* = .19. We analysed the LS-S intercorrelations with the given mental health outcomes and sociodemographic risk factors by Spearman correlations and Chi-square tests. Correlation coefficients of the LS-S were overall comparable to the single-item measure of loneliness. Chi-square test results indicated significant positive associations of both loneliness measures and sex and partnership. Further, both loneliness measures showed negative correlations with household size. Also, significant negative correlations between loneliness and domain-specific FLZ scores were observed in both cases. However, correlations of loneliness based on the single-item measure with PHQ-2 scores and GAD-2 scores were smaller than correlations observed of the LS-S with PHQ-2 scores and GAD-2 scores. For details, see Table 4.

**Table 4:** Associations between the single-item measure of loneliness and the LS-S with other mental health outcomes and previously established sociodemographic risk factors.

	Loneliness	
	Single-item measure of loneliness	LS-S
Mental health and well-being		
*Loneliness (single-item measure of loneliness)*		
*Loneliness (LS-S)*	.57***	
*PHQ-2*	.41***	.56***
*GAD-2*	.34***	.51***
*FLZ- Friends and acquaintances*	-.21***	-.30***
*FLZ- Hobbies and leisure*	-.20***	-.31***
*FLZ- Health*	-.22***	-.29***
*FLZ- Financial security*	-.24***	-.33***
*FLZ- Job*	-.24***	-.32***
*FLZ- Housing*	-.18***	-.28***
*FLZ- Family life and children*	-.23***	-.26***
*FLZ- Partnership and sexuality*	-.28***	-.33***
Sociodemographic risk factors		
*Age*	.03*	-.01
*Sex*	.09***	.08***
*Household size*	-.13***	-.11***
*Partnership*	.19***	.19***

*Note*. LS-S = Loneliness Scale-SOEP; correlation coefficients based on Spearman correlation; Associations between the single-item measure of loneliness and sex and partnership were analysed by Χ^2^-tests

* *p* < .05

**p < .01

***; *p* < .001.

## Discussion

Given the importance of loneliness as a risk factor for physical and mental health and the need for its economical assessment (e.g., in large-scale surveys and cohort studies), the aim of the present research was to investigate a single-item screening measure of loneliness based on two representative population samples.

In a combined sample of two German representative population samples covering a large age range from 16 to 96 years, 23.4% of participants reported some degree of loneliness, 3.4% among them severe loneliness. The highest rates of loneliness were found in women and in participants in early adulthood and older than 74 years. Among women, severe loneliness was more frequent in the oldest age group, while men reported the highest rates in midlife. Descriptive comparisons of the overall prevalence of loneliness based on the single-item measure of loneliness and the well-established LS-S showed great similarities. The majority of the participants were not classified as lonely on both measures. Moderate correlations were found between the single-item measure of loneliness and all three LS-S items. The strongest relation (*ρ* = .55, *p* < .01) was observed with the item "absence of companionship", which comes closest to the definition of loneliness in the single-item measure as being distressed by a lack of social contact. Moderate positive correlations with depression symptoms and anxiety symptoms and a moderate negative correlation with satisfaction with life as well as correlations with demographic risk factors were overall comparable to correlations of the LS-S and mental health outcomes resp. sociodemographic risk factors and indicated acceptable construct validity.

Overall, the reported prevalence rates are in accordance with previous findings on loneliness in the general population [21, 30, 45]. However, some studies indicated a lower prevalence of loneliness in midlife (7.7% to 12.0%) [46]. Lower degrees of loneliness were also previously found in the GHS, a large, prospective community cohort study in mid-Germany which used the single-item measure for loneliness. Beutel et al. [2017] found that a total of 10.5% of participants reported some degree of loneliness; 1.7% indicated severe distress by feeling lonely. The higher prevalence of loneliness observed in the present sample may be the result of actual differences between samples, over time, or regions. In fact, in the present study, the highest loneliness rates were reported by young participants aged 25 to 34 years, and by the oldest participants (aged above 74 years). Notably, the GHS wave studied did not include these two at-risk age groups. Furthermore, both surveys from which we drew our data were conducted during the first lockdown period (from March to July 2020) and during a further period of severe restrictions, including home-schooling, working from home, and restrictions regarding the number of non-household members allowed to meet (November 2020 to May 2021) in Germany in the context of COVID-19 pandemic. Beutel et al. [2017] used data that had been collected from 2007 to 2012. As loneliness has also been discussed as a major psychosocial consequence of the COVID-19 pandemic, higher degrees of loneliness in the present work may be influenced by the pandemic or more general increases over time. However, recent findings on whether loneliness has increased overall since the pandemic started, are inconsistent. A recent review with meta-analysis showed increases in prevalence rates of loneliness in the pandemic context [5], while other studies found only evidence of an increase in loneliness in younger age groups under 30 years [30]. Regional differences may be another explanation for the higher prevalence of loneliness in the present sample. Lampert and Koch-Gromus [47] argued that significant regional differences regarding social parameters such as unemployment and poverty exist within Germany, which contribute to disparities in terms of health and life expectancy. Therefore, it should be noted that the GHS as a population-based cohort study of the metropolitan Rhine-Mine region in western Mid-Germany may constitute a wealthier and more homogenous sample with a higher level of education compared to the nationwide survey sample used in this work.

In accordance with recent studies [9, 14, 20, 21], we found higher levels of loneliness among women compared to men. Although other investigations revealed reverse results with higher rates of loneliness among men [22, 23]. As suggested by Nicolaisen and Thorsen [2014], the observed sex differences in the present study may be the result of using a more direct measure by direct asking about the subjective experience of loneliness on the single-item measure. Similar to previous studies [3, 4, 19], we observed that loneliness is more prevalent in younger age groups and the oldest age group >75 years. We further identified different patterns of loneliness when considering sex and age groups in line with previous findings [14, 21]. Among women, loneliness was highest in the oldest group, while men reported the highest degrees of loneliness in midlife. Higher vulnerability among older women may be explained due to their longer life expectancy. Therefore, women had a higher risk of being widowed or of struggling with functional decline, both of which are relevant circumstances in older age associated with loneliness [9, 18]. The peak of loneliness in middle-aged men may be indicative of midlife crises [48]. Levels of loneliness were considerably higher among participants without a partner. Women not living in a partnership reported the highest rates in those aged above 74 years, suggesting a potential explanation of higher vulnerability among older women due to widowhood. The highest degrees of loneliness in men were found in the age group of 55 to 64 years, similar to the overall prevalence stratified by sex and age group.

In order to compare the prevalence rates of loneliness calculated on the basis of the two loneliness measures, we created two binary variables. To dichotomize loneliness based on the single-item measure of loneliness a score of greater than 1 was used to define the presence of loneliness. Like other researchers [36], we used scores in the top quintile to classify participants as “not lonely” and “lonely” on the LS-S. Overall, rates of loneliness were quite similar on both measures. About one in four participants reported loneliness (single-item measure of loneliness: 23.5%; LS-S: 25.1%). The minimally higher prevalence rates on the LS-S might stem from the single-item measure of loneliness being a more “direct”, by inquiring whether people are often alone or have few contacts, measure while the LS-S captures the distressing feeling of loneliness in a more indirect way [49]. This also applies to the wording of the response options which in the case of the single-item measure of loneliness indicate different *levels of distress*, whereas respondents are asked to rate the *frequency* of different experiences. These differences in both measures may also account for the moderate convergent validity (*ρ* = .57, *p* < .001) [50]. Further, correlations of the three LS-S items with the single-item measure of loneliness varied across the scale’s items, the closest relation was found with the Item “absence of companionship” (*ρ* = .55, *p* < .010). This is what we expected to find because the focus of the item is directly on companionship, most similar to the wording of the single-item measure of loneliness. The single-item measure of loneliness is suitable for screening for loneliness when situational constraints limit the use of longer instruments (e.g., large-scale surveys, limited time, cost considerations). The single-item measure of loneliness may be also more suitable for vulnerable persons (e.g., the elderly), and persons who lack the motivation or cognitive resources to fill out a long questionnaire.

Intercorrelations of the single-item measure of loneliness with depression symptoms, anxiety symptoms, and satisfaction with life were analysed in order to determine its construct validity. As in previous studies, we found moderate positive associations with depression symptoms and anxiety symptoms and a negative association with satisfaction with life [20, 21, 25], supporting the construct validity of this single-item measure. Further, construct validity was provided by the association of the single-item measure of loneliness with established demographic risk factors for loneliness such as household size and partnership. All intercorrelations were comparable to correlations of the LS-S and mental health outcomes or sociodemographic risk factors, respectively. Merely correlations of loneliness based on the single-item measure of loneliness with depression and anxiety were smaller. Altogether, the results supported the construct validity of the single-item measure of loneliness.

Lastly, the present investigation yields evidence to place the single-item measure of loneliness in the context of other loneliness measures that have been previously and are currently used in international research endeavors: Following the taxonomy proposed by Valtorta et al. [2016] [51], the single-item measure of loneliness allows an ascertainment with a low degree of subjectivity in terms of actually available social connections, i.e., participants are asked about their degree of involvement with others. At the same time, the single-item measure of loneliness captures subjective distress associated with this report, which fills a gap in the existing measures. Especially in the wake of the COVID-19 pandemic, there is an urgent need to monitor loneliness and its physical and mental health sequelae in the population. To this end, large-scale population studies are required. In the context of these efforts, the *brevity* of the included assessments is crucial, both to control costs and limit participant burden. The present work presents a validated single item that is ideally suited to be included in these timely surveys, or as an addition to existing prospective cohorts that combine different modes of assessment (e.g., physical examinations, laboratory studies).

## Strengths and limitations

The use of two representative population samples is a strength of the study. Therefore, findings are unbiased regarding age, sex, and level of education, which have previously all been shown to impact on loneliness in the population. Besides these strengths, some limitations should also be considered. First, we concede, that we used no validated cut-off score to define loneliness on the LS-S. As no single established cut-off score exists for the LS-S, in line with previous studies, we used scores in the top quintile to define loneliness (in analyses using a dichotomized version of the measure). We were not able to validate its diagnostic accuracy by using an external reference (as possible with e.g., diagnoses of major depressive disorder when validating a depression measure). Therefore, the presented results should be interpreted with caution. Furthermore, we used an indirect measure of loneliness to assess the convergent validity of the more direct single-item measure of loneliness, although previous research has pointed out differences between direct and indirect measures in identifying lonely people [22, 52].The results of this study are primarily generalizable to the general population. Psychometric properties may change when the single-item measure is applied to different samples, e.g., clinical populations. Lastly, in the present study, mental distress was measured using short screening tools (owing to the restrictions associated with large population surveys).

Despite the growing evidence for good psychometric properties of single-item measures, many researchers still view them critically [28]. This study provides evidence that a single-item measure can be a valid and economical screening measure, especially in large-scale population studies, and it presents such a measure that could be used by others aiming to build on this work. Future research could expand the present results by further scrutinizing its validity and reliability, in particular regarding domains we were not able to address with the present study design, such as test-retest-reliability, predictive validity and associations with other constructs, e.g., in a nomological net using a wider variety of measures, especially multi-item measures of loneliness. To extend validation efforts beyond self-reports, it will be useful to examine the convergence between self- and informant-ratings of loneliness [29]. The psychometric properties and performance of single-item measures is also a crucial issue in intensive longitudinal research designs that have to rely on very short/single-item assessments, see e.g. Ernst, Tibubos [53].

## Conclusion

A brief, yet high-quality assessment of loneliness is crucial to monitor its prevalence as well as potential changes over time in the population and to yield reliable results regarding its effects on mental health outcomes. Against this background, the present work aimed to provide a validation study of a new single-item measure of loneliness. Overall, the results supported its validity and reliability, e.g., based on descriptive comparisons of prevalence rates calculated based on the single-item measure of loneliness and an established three-item scale. Thus, the single-item measure of loneliness could constitute a useful, informative screening measure (e.g., large-scale surveys). Its psychometric properties and performance should be further evaluated by future research.

## Supporting information

S1 File Items included in the surveys. The surveys were conducted in German. An English translation/the authorized English version of the respective items is only provided as a courtesy to readers of this work.
(DOCX)

S2 File (SAV)

S3 File (SAV)
