# Supplementary material for: Screening for loneliness in representative population samples: Validation of a single-item measure
Source: PLoS One. 2023 Mar 16;18(3):e0279701. doi: 10.1371/journal.pone.0279701 (PMC10019616; doi:10.1371/journal.pone.0279701)
Supplement: S1 File — The surveys were conducted in German. An English translation/the authorized English version of the respective items is only provided as a courtesy to readers of this work. (DOCX) [file pone.0279701.s001.docx]

Items included in the surveys. Note: The surveys were conducted in German. An English translation/the authorized English version of the respective items is only provided as a courtesy to readers of this work.

1. **GHS-item**

German version:

Trifft folgendes auf Sie zu und wenn ja, wie stark belastet Sie das?

|  | Nein, trifft nicht zu. | Ja, trifft zu, aber hat mich nicht belastet. | Ja, trifft zu, aber hat mich wenig belastet. | Ja, trifft zu, aber hat mich mittelmäßig belastet. | Ja, trifft zu, aber hat mich stark belastet. |
| --- | --- | --- | --- | --- | --- |
| Häufiges Alleinsein, zu wenig Kontakte |  |  |  |  |  |

English version:

Does the following apply to you and if yes, how much do you suffer from it?

|  | No, does not apply. | Yes, it applies, but I do not suffer from it. | Yes, it applies, and I suffer slightly. | Yes, it applies, and I suffer moderately. | Yes, it applies, and I suffer strongly. |
| --- | --- | --- | --- | --- | --- |
| I am frequently alone/have few contacts |  |  |  |  |  |

1. **Loneliness scale-SOEP**

German version:

Wie oft haben Sie das Gefühl…

|  | Nie | Selten | Manchmal | Oft | Sehr oft |
| --- | --- | --- | --- | --- | --- |
| …dass Ihnen die Gesellschaft anderer fehlt? |  |  |  |  |  |
| …außen vor zu sein? |  |  |  |  |  |
| …dass Sie sozial isoliert sind? |  |  |  |  |  |

English version:

How often do you feel…

|  | Never | Rarely | Sometimes | Often | Very often |
| --- | --- | --- | --- | --- | --- |
| …that you lack companionship? |  |  |  |  |  |
| …left out? |  |  |  |  |  |
| …isolated from others? |  |  |  |  |  |

1. **PHQ-2**

German version:

Wie oft fühlten Sie sich im Verlauf der letzten zwei Wochen durch die folgenden Beschwerden beeinträchtigt?

|  | Überhaupt nicht | An einzelnen Tagen | An mehr als der Hälfte der Tage | Beinahe jeden Tag |
| --- | --- | --- | --- | --- |
| Wenig Interesse oder Freude an Ihren Tätigkeiten |  |  |  |  |
| Niedergeschlagenheit, Schwermut oder Hoffnungslosigkeit |  |  |  |  |

English version:

Over the last two weeks, how often have you been bothered by the following problems?

|  | Not at all | Several days | More than half the days | Nearly every day |
| --- | --- | --- | --- | --- |
| Little interest or pleasure in doing things |  |  |  |  |
| Feeling down, depressed, or hopeless |  |  |  |  |

1. **GAD-2**

German version:

Wie oft fühlten Sie sich im Verlauf der letzten zwei Wochen durch die folgenden Beschwerden beeinträchtigt?

|  | Überhaupt nicht | An einzelnen Tagen | An mehr als der Hälfte der Tage | Beinahe jeden Tag |
| --- | --- | --- | --- | --- |
| Nervosität, Ängstlichkeit oder Anspannung |  |  |  |  |
| Nicht in der Lage sein, Sorgen zu stoppen oder zu kontrollieren |  |  |  |  |

English version:

Over the last two weeks, how often have you been bothered by the following problems?

|  | Not at all | Several days | More than half the days | Nearly every day |
| --- | --- | --- | --- | --- |
| Feeling nervous, anxious or on edge |  |  |  |  |
| Not being able to stop or control worrying |  |  |  |  |

1. **Fragebogen zur Lebenszufriedenheit (FLZ) / Questionnaire on Life Satisfaction**

German version:

Bei den folgenden Fragen geht es darum, wie zufrieden Sie mit Ihrem Leben und mit den einzelnen Aspekten Ihres Lebens sind.

Bitte kreuzen Sie an, wie zufrieden Sie in den einzelnen Lebensbereichen sind.

Wie zufrieden sind Sie…

|  | Unzufrieden | Eher unzufrieden | Eher zufrieden | Ziemlich zufrieden | Sehr zufrieden |
| --- | --- | --- | --- | --- | --- |
| Freunde / Bekannte |  |  |  |  |  |
| Freizeitgestaltung / Hobbys |  |  |  |  |  |
| Gesundheit |  |  |  |  |  |
| Einkommen / finanzielle Sicherheit |  |  |  |  |  |
| Beruf / Arbeit |  |  |  |  |  |
| Wohnsituation |  |  |  |  |  |
| Familienleben / Kinder |  |  |  |  |  |
| Partnerschaft / Sexualität |  |  |  |  |  |

English version:

The following questions are about your satisfaction with your life and with the individual aspects of your life.

Please tick how satisfied you are in each area of your life.

How satisfied are you...

|  | Not at all satisfied | Rather dissatisfied | Rather satisfied | Quite satisfied | Extremely / very satisfied |
| --- | --- | --- | --- | --- | --- |
| Friends / acquaintances |  |  |  |  |  |
| Leisure time / hobbies |  |  |  |  |  |
| Health |  |  |  |  |  |
| Income / financial security |  |  |  |  |  |
| Occupation / work |  |  |  |  |  |
| Housing / living conditions |  |  |  |  |  |
| Family life / children |  |  |  |  |  |
| Partner relationship / sexuality |  |  |  |  |  |
